# Comparison of Fascin Expression in Oral Verrucous Carcinoma and Oral Squamous Cell Carcinoma

**DOI:** 10.1155/ijod/5530533

**Published:** 2025-05-06

**Authors:** Nima Mohammadi, Shadi Saghafi Khadem, Saghar Emami Ardestani, Mohammad Hossein Nikbakht

**Affiliations:** ^1^Dental Research Center, Department of Periodontics, School of Dentistry, Isfahan University of Medical Sciences, Isfahan, Iran; ^2^Department of Oral and Maxillofacial Pathology, School of Dentistry, Mashhad University of Medical Sciences, Mashhad, Iran; ^3^Student Research Committee, School of Dentistry, Isfahan University of Medical Sciences, Isfahan, Iran

**Keywords:** fascin, immunohistochemistry, oral squamous cell carcinoma, pathology, verrucous carcinoma

## Abstract

**Introduction:** One of the diagnostic problems of pathology is to differentiate between oral verrucous carcinoma (OVC) and oral squamous cell carcinoma (OSCC). Fascin increases the invasion of normal and neoplastic cells by stabilizing cytoplasmic filamentous actin. The present study aimed to investigate the expression of fascin in OSCC and OVC.

**Methods:** This descriptive-analytical cross-sectional was conducted on 25 blocks of OSCC, 22 blocks of OVC, and 10 blocks of healthy mucosa as a control group. After immunohistochemical staining, samples were observed by two maxillofacial pathologists simultaneously, and the percentage of stained cells, intensity of staining, and the location of stained cells were obtained.

**Results:** There was no significant difference in the gender (*p*=0.123) and age (*p*=0.276) distribution of participants in the groups. There was a significant difference in the distribution of the involved area in the patients of the studied groups (*p* < 0.001). There was a significant difference in the intensity of staining and the percentage of stained cells between the studied groups (*p* < 0.001).

**Conclusions:** The percentage and intensity of staining were higher in the OSCC, OVC and, control groups, respectively. It seems that Fascin expression has an important role in predicting OVC and OSCC.

## 1. Introduction

Oral squamous cell carcinoma (OSCC) is the most common malignant tumor in the oral cavity and the sixth most common malignancy worldwide, with 300,000 new cases in a year [1]. This neoplasm develops from the mucosal epithelium in the oral cavity with varied differentiation [2]. Despite various advanced treatment methods, only 60% of patients survive for over 5 years. Early diagnosis of this tumor plays a significant role in increasing the survival rate of patients [3].

Oral verrucous carcinoma (OVC) is a rare, better-differentiated type of SCC with morphologic traits of broad-based squamous epithelial proliferation and prominent keratinization. Less than 1%–10% of OSCCs are OVC. OVC grows locally, rarely metastasizes, and has a better prognosis than other OSCCs (with an overall 5-year survival rate of 77%–86%) [1, 2]. One of the diagnostic problems of pathology is to differentiate between OVC and an invasive OSCC, and sometimes, to rule out OSCC, many OVC samples must be cut several times or the patient must have surgery again [4]. Consequently, studying the capability of biologic markers for differential diagnosis can represent a leap.

Fascin is a protein localized in specific cell protrusions (filopodia and invadopodia), which are the dynamic areas of cell movement direction and play a significant role in matrix interactions. This protein organizes actin bundles in different cell communities and forms actin ridges on the cell surface and cytoplasmic structures. These structures are important in cell-to-cell communication, cell migration, and cellular matrix adhesion. Fascin protein is essential for thrombospondin-1 migration and increases the invasion and migration of normal and neoplastic cells by stabilizing cytoplasmic filamentous actin [5–7].

Fascin protein is a determining protein in cell mortality and adhesion, and recently the increase in fascin occurrence in both mRNA and protein levels is a determining indicator concerning metastasis to neck lymph nodes or poor prognosis in epithelial cancers such as breast, skin, colon, ovarian, and lung carcinomas has been reported [8, 9]. The exhibition of fascin is related to the invasion of invasive tumors and has recently been considered a predictive marker and a therapeutic target for metastatic diseases [9]. Fascin immunoreactivity in the areas of the invasion zone of different tumors shows that staining with this marker can be valuable for better-detecting surface invasion sites or primary stromal invasion [5, 6]. A statistically significant association between an increase in tumor stage, lymph node metastases, decreased differentiation, increased recurrence, and reduced patient survival has been observed with fascin expression in OSCC [10–12].

OVC and OSCC are different in metastatic and invasive behavior, and fascin is a protein important in invasion. Various studies have shown that fascin can detect areas of active invasion [11, 13, 14]. It could be a major diagnostic step if there is a significant difference in fascin expression between OSCC and OVC. Regarding different molecular signatures, prognosis, and invasiveness of OVC and OSCC, and as no study has been done on the diagnostic value of this marker in VC, the present study aimed to investigate the expression of fascin in OSCC and OVC.

## 2. Materials and Methods

### 2.1. Ethical Approval and Study Design

This descriptive-analytical cross-sectional study was approved by the Research Ethics Committee of Mashhad University of Medical Sciences (IR.mums.sd.REC.13940119).

### 2.2. Participants

The study was conducted on 25 paraffin blocks of OSCC, 22 paraffin blocks of OVC, and 10 paraffin blocks of healthy mucosa adjacent to fibrotic lesions (as a control group) from patients referred to the pathology department of the Faculty of Dentistry of Mashhad University of Medical Sciences (57 in total). The lesions were accurately diagnosed based on clinicopathology criteria by two maxillofacial pathologists simultaneously. Pathologists were professor of Mashhad University of Medical Sciences and experienced in immunohistochemistry evaluation and the diagnosis of OSCC and OVC

OVC is diagnosed based on consisting of filiform projections covered with thick, well-differentiated keratinized squamous epithelium, comprising one to a few layers of basal cells, and numerous spinous cells without cytological abnormalities. Stromal invasion with a well-defined margin is present in this lesion [15]. For diagnosing OSCC, various degrees of squamous differentiation with islands and cords of tumor cells (pink-stained cytoplasm, intercellular bridges, and keratinization and pearl formation) were important. Invasive growth is the most prominent disease and pathological feature of OSCC. The lesion epithelium appears to be irregular and penetrates through the basal membrane with apparent invasion into the subepithelial connective tissue layer or even deeper tissues. Chronic inflammatory diseases and interstitial fibrosis are often associated with it [16].

Paraffin blocks that had a sufficient amount of the studied sample tissue for immunohistochemical staining and confirming the diagnosis were included, and the controversial diagnosis or distorted paraffin blocks that do not have enough tissue were excluded from the study.

### 2.3. Setting

At first, according to the information available in the archive, the demographic characteristics of the selected patients, including age, sex, and location of the lesion, were recorded.

From each of the selected paraffin blocks, a four-micron section was prepared for immunohistochemical staining to check the incidence of the fascin immunohistochemical marker. Two pathologists examined the stained slides without knowledge of the type of lesion using a binocular optical microscope (Labomed, USA) with magnifications of 100 and 400. The slides were stained with hematoxylin and eosin, and then the samples were subjected to immunohistochemical staining for fascin (Monoclonal MxH Fascin, Clone 55k-2).

The staining technique for the samples was done as follows:

The slices were placed on the active slide, and the sample was placed in the oven for 15 min at 60°C to be fixed. Then, the samples were placed in xylenol solution for deparaffinization, which included 4xylenol solutions, and the sample was placed in each solution for 5 min. The samples were washed with water and placed in distilled water for 2 min. In the next step, Dako buffer was used for antigen retrieval for 30 min. The lid of the container was closed and it was placed at a temperature of 94°C–98°C. After cooling the solution at room temperature, we removed the samples from the solution and washed them with distilled water. In the next step, the samples were placed in Tris buffer (pH = 7.6) and after 3 min in oxygenated water to inhibit hydrogen peroxide inside the cell (5% hydrogen peroxide for 10 min) and again for 5 min in Tris buffer. From this point, the inside of the container was wet. The primary antibody (fascin) was added to the samples for 1.5 h. Then it was placed in the Tris buffer for 5 min. Then we added the secondary antibody linked to the HPR enzyme for half an hour and put it in the Tris buffer again for 5 min. After that, we added one drop of brown kerogen solution (combined with 1 cc of kerogen buffer with 1 drop of DAB) to the samples for 10 min and then washed them with distilled water for 5 min. We added Mayer's hematoxylin for 2 min to color the background and washed it again with distilled water. In order to extract water from the tissue, the samples were placed in different degrees of alcohol (from 70% to pure alcohol) because the water that entered into the tissue must be removed gradually. Finally, the samples were placed in xylenol solution, dried, and covered glass attached.

**Figure 1:**
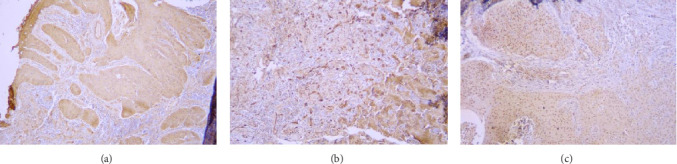
Photomicrograph shows OSCC samples. (A) Staining of grade 1 OSCC with weak intensity and 25%–50% of stained cells (×100). (B) Staining of grade 2 OSCC with weak intensity and 50%–75% of stained cells (×400). (C) Staining of grade 1 OSCC with moderate intensity and 50%–75% of stained cells (×400).

**Figure 2:**
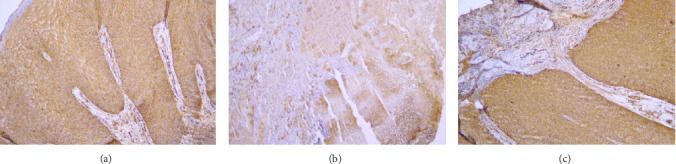
Photomicrograph shows OVC samples (A) Staining with moderate intensity and 50%–75% of stained cells (×400). (B) Staining with moderate intensity and 25%–50% of stained cells (×400). (C) Staining with severe intensity and 50%–75% of stained cells (×400).

Samples with different stained cell percentages and stain intensities with 100 and 400 magnifications are presented in Figures 1 and 2.

### 2.4. Data Measurement

In the microscopic examination, the samples in which the cells were positive in terms of immunohistochemical staining were determined with 40 and 100 magnifications, and in these samples, the areas with the highest concentration of cells (HOT spot) were selected. To count the stained cells, the slides were examined with a low-power field (LPF) and the counting was done in five areas rich in stained cells and the average was taken. Cells showing cytoplasmic staining were considered positive. If less than 5% of cells were stained, number 0, if 5% and 25% of cells were stained, number 1, between 25% and 50% of cells were stained, number 2, between 50% and 75%, number 3, and more than 75%, number 4 was considered. In terms of the staining intensity, the same cells were examined; weak staining was given as 1, moderate as 2, and strong as 3 [17]. Also, in terms of the location of stained cells, if only the basal cells were stained, the number 1, all the cells below the surface layer were stained, the number 2, and if the cells were stained the entire thickness of the epithelium, the number 3 was recorded [18].

### 2.5. Study Size

Samples were selected by the easy sampling method. The following formula was used to determine the sample size in each group based on El-Rehim et al. study [19], assuming the number of samples in each group was equal (*α* = 0.05, *p* [The percentage of dyeability] = 83%, *d* = 0.13).

*n* = $\frac{Z1-\alpha /2*p*q}{d2}.$

In this study, 60 samples were used (25 for each group and 10 for the control group). It should be noted that three samples related to VC were removed due to problems related to coloring.

### 2.6. Statistical Methods

The data obtained in this study were entered into SPSS version 16 software. To describe the data, appropriate tables and graphs were used to show the dispersion and central tendency indicators, and in the data analysis, chi-square, Fisher's exact, independent *t*, ANOVA, and Kruskal–Wallis statistical tests were used. The significance level was considered *α* = 0.05.

## 3. Results

The sample comprised 57 paraffin blocks, which were in three groups: OSCC, OVC, and control, with numbers of 25, 22, and 10, respectively. The paraffin blocks of OSCC consisted of 12 grade 1 OSCC, 9 grade 2 OSCC, and 4 grade 3 OSCC.

### 3.1. Demographic Data

Demographic data and the area of involvement in the groups are shown in Table 1. There were 9 (40.9%) and 14 (56%) females in the OSCC and OVC groups, respectively. Females comprised 66.7% of the grade 1 OSCC group, while in grades 2 and 3, females comprised 22.2% and 25% of patients, respectively. The chi-square test was not significant regarding the gender distribution of people in the four groups (*p*=0.123).

The average age of OVC group patients was 64.54 ± 13.29 and SCC group patients were 62.32 ± 15.43. There was a higher mean age in grade 1 OSCC patients (68.0 ± 9.94) compared to grade 2 (56.67 ± 16.23) and grade 3 (58.0 ± 24.32) OSCC patients. According to ANOVA test, there was no significant difference between the four groups in age (*p*=0.276).

The most common areas of involvement in OVC patients were the mandibular vestibule (5 patients, 22.7%) and the floor of the mouth (5 patients, 22.7%). In patients with OSCC, the most common location was the tongue (11 patients, 44%). The tongue was the most common affected area in both the grade 2 and grade 3 OSCC group patients. Fisher's exact test was significant in comparing the distribution of the involved area among the patients in the four groups (*p* < 0.001).

### 3.2. Percentage of Stained Cells

Data showing the percentage of stained cells in the samples is given in Tables 2 and 3. The Kruskal–Wallis test was significant regarding the comparison of the percentage of stained cells between the studied groups (*p* < 0.001). In 12 cases (54.55%) of the OVC samples and 18 cases (72%) of the SCC samples (7 grade 1, 8 grade 2, and 3 grade 3), the percentage of stained cells was more than 75%.

### 3.3. Intensity of Staining

The intensity of staining in the samples is given in Tables 4 and 3. In OVC cases, the highest intensity of staining was related to intensity 2 (13 cases, 59.09%), and the lowest level of intensity was related to intensity 3 (one case, 4.55%). In OSCC cases, the highest intensity of staining was related to intensity 3 (12 cases, 48%), and the lowest level of intensity was related to intensity 1 (one case of grade 2 OSCC, 4%). The control samples had an equal amount (5 cases) of severity 1 and 2, and none of them showed severity 3. Among the three grades of OSCC, the grade 2 and grade 3 groups had the higher frequency of intensity 3 staining, whereas the grade 1 group showed intensity 2 staining more frequently. The Kruskal–Wallis test was significant for comparing the intensity of staining between the studied groups (*p* < 0.001).

### 3.4. Location of Stained Cells

In OVC cases, stained cells were observed in the entire thickness of the epithelium in 8 cases, beneath the surface layer in 11 cases, and only in the basal cells in 3 cases. In OSCC cases, in 19 cases, the cells were present in the entire thickness of the epithelium, and in 6 cases, the stained cells were under the surface layer. In the control group, in 4 cases the cells were under the surface layer, and in 6 cases the cells were in basal cells.

## 4. Discussion

This study aimed to investigate the expression of fascin in OVC and OSCC and its capability to be used as a diagnostic tool. The results of our study showed a significant difference in the intensity of staining and the percentage of stained cells, which decreased from samples with higher-grade OSCC to samples with OVC and control samples, which confirmed the increase in fascin protein expression in OSCC patients.

Fascin is an actin-binding protein that increases the invasion and migration of normal and neoplastic cells by stabilizing cytoplasmic filamentous actin and plays a role in cell motility [5–7]. Upregulation of fascin expression occurs in many human carcinomas [10, 20–24]. In a study by Shimamura et al. [25] in 2011, it was reported that fascin expression was significantly higher in dysplasia and oral carcinoma in situ (CIS) compared to benign diseases, including papilloma. In their study, all samples of invasive SCC (6/6) showed fascin expression in the cytoplasm, extending from the basal layer to the superior layer of the epithelium. They conclude that fascin may have a role in the early stage of carcinogenesis. Zhang et al. [26] in 2006 reported that the degree of fascin expression and rate of overexpression progressively increase in the development from normal epithelium to simple hyperplasia, dysplasia, CIS, and finally invasive esophageal squamous cell carcinoma. Therefore, the increase in the expression of this marker in SCC and then VC samples in our study is not far from expected and suggests that fascin can be used in the accurate diagnosis of SCC and VC cases.

Contrary to the results obtained from our study, in the Alam et al. [10] study in 2012, which was conducted to identify the role of fascin in the progression of OSCC, fascin protein was not found in 25.19% of cases, 41.98% of samples had weak expression, and only 32.28% of cases showed increased expression of this protein. Consistent with the result of the above study, Lee et al., in their study of the role of fascin in OSCC progression, reported that 20 of 40 samples of OSCC showed negative to low expression of fascin.

The possible causes of the mentioned difference can be related to different stages of performing IHC staining, eye count, and also the volume of different samples. Technical errors during immunohistochemistry staining, such as suboptimal antigen retrieval due to insufficient time or temperature, can mimic the weak expression view. Also, errors in incubation time or temperature and improper degradation of antigen may be the reasons for differences.

In our study, the stained cells in 11 of 22 OVC cases were under the surface layer and in eight cases, the stained cells were present in the entire thickness of the epithelium. Whereas of 25 OSCC cases, in 19 cases staining was present in the entire thickness of the epithelium, and in six cases the stained cells were under the surface layer. Interestingly, in the control group, none of the cases had fascin expression in the entire thickness. These results indicated that fascin-expression degrees, based on the thickness of the epithelium expressing fascin, increase as the aggressiveness and invasion of cells increase. It is consistent with the results of Shimamura et al.'s [25] study, in which 6 of 7 cases with dysplasia and all cases of CIS showed fascin expression throughout the whole epithelium.

In 2007, Chen et al. [20] reported upregulation in the expression of fascin in most of their OSCC samples, with variable and heterogeneous expression in the tumor parts, varying from mild to severe and from localized to diffuse. They observed in the majority of patients with OSCC intense reactivity at the margins of invasive fronts of the individual tumor. Chen et al. found that both intensity and distribution of fascin expression were correlated with tumor size and clinical classification. They concluded that the expression of the fascin protein may play an important role in predicting OSCC. Similarly, our results showed that higher intensity and percentage of staining were observed in most of the cases with higher grades of OSCC. Although the intensity and percentage of fascin marker staining in OSCC were investigated in the above study, the classification, especially in terms of staining percentage, was different from our study, and samples with VC were not evaluated in this study.

In Papaspyrou et al.'s [18] study in 2014, which 25 patients were investigated, a longer median overall survival was observed in the group with low fascin expression (54 months) compared to the high fascin group (38 months), but the difference was not statistically significant. Consistent to Papaspyrou et al.'s observation, Wu et al. [27] found that in nasopharyngeal SCC, the overall survival and disease-free survival rates were lower for individuals with higher levels of fascin expression.

In the study of Lee et al. [11] in 2007 regarding the high expression of fascin with the invasion of OSCC, they found that the high expression of fascin in clinical samples of OSCC was significantly associated with tumor recurrence and overall patient survival. Similar to the results of Wu et al. [27] and Papaspyrou et al. [18], a longer mean overall survival was observed in patients with lower fascin expression. As a result, these studies indicated the capability of fascin used as a prognostic tool in OSCC, which is in contrast to the studies of Zhang et al. [26] in esophagus SCC and Öztürk et al. [28] in laryngeal SCC, in which no significant association was observed between fascin expression and prognostic factors.

All cases of OVC and OSCC in our study had fascin stromal expression. Stromal invasion with a well-defined margin is present in OVC [15]. Similarly, in OSCC, epithelium penetrates through the basal membrane with apparent invasion into the subepithelial connective tissue layer or even deeper tissues [16]. Regarding this, the observed stromal expression of fascin is not far from expected. Fascin expression in the stroma could be due to endothelial cells and fibroblasts, which normally express fascin [29]. Another possible reason contributing to this observation is cancer-associated fibroblasts (CAFs), which play a role in the microenvironment by promoting tumorigenesis and metastasis [30]. The CAFs can be derived from endothelial cells, local fibroblasts, and tumor epithelial cells by epithelial–mesenchymal transition (EMT) [31]. Based on the Chen et al.'s [32] study, fascin protein may play an important role in causing EMT. Our observation is consistent with Rodrigues et al. [33] that observed fascin expression in inflammatory cells and endothelial cells in the stroma of OSCC cases.

Fascin is normally present in vascular endothelial cells and fibroblasts [29], and low levels of fascin have been reported in stratified basaloid squamous cells in the esophagus [22]. Shimamura et al.'s study [25] showed that fascin is not expressed in the normal oral epithelium or only weakly expressed in basal cells, but in Papaspyrou et al.'s [18] study, 7 out of 19 samples with normal epithelium (healthy epithelial tissue adjacent to the tumor) showed a score of 2. In our study, the staining intensity score was 2 in 5 samples out of 10 control samples. The reason for this could be that the epithelium may have been obtained from the vicinity of the tumor and therefore may have the same pathological conditions as the tumor and the same increased expression. The epithelium may be normal at the macroscopic level and under the light microscope but have structural changes at the molecular level. Also, upregulation of fascin expression might be an expression pattern specific to tissue or a correlation between fascin and cell proliferation capability.

Zhang et al. [26] found that proliferative activity of the cell led to an increase in fascin expression. Also, Alam et al. [10] reported that the proliferation of cells with fascin overexpression increased. Previous studies suggested several possible mechanisms for fascin such as a decrease in E-cadherin and *β*-catenin levels leading to disorganization of cell–cell contacts [10, 11, 23] and an increase in extracellular matrix proteases digesting the basement membrane, such as MMP-2 and MMP-9, which facilitate invasion [10, 34]. Regarding this, inhibition of fascin could be a therapeutic target for OVC and OSCC.

Some other studies investigated the capability of other biomarkers to diagnose OVC and OSCC. Angadi and Angadi [35] studied the expression level of GLUT-1, which was observed in 25%–50% of patients with OSCC and more than 50% of OVC cases. GLUT_1, which regulates energy metabolism, was reported to gradually increase from normal epithelium to OSCC and OVC. Some earlier studies reported significant differences in MMP-2 and MMP-9 in OVC and all OSCC grades [36, 37]. However, the differentiation between low-grade OSCC and OVC is still a concern.

Table 5 presents an overview of some literature findings mentioned in the discussion.

To the best of our knowledge, this is the first study comparing fascin expression between OVC and OSCC. We observed a significant difference in staining intensity and percentage of stained cells, which was higher in OSCC compared to OVC. Further investigations are recommended to examine the effectiveness of different biomarkers or combinations of them as diagnostic tools for OSCC and OVC.

Due to the low sample size, we would not be able to investigate factors such as smoking, chewing tobacco, alcohol, chemotherapy, and radiation therapy. Although, according to Papaspyrou et al.'s [18] and Lee et al.'s [11] studies, the fascin expression was not associated with alcohol consumption and smoking in OSCC, chewing tobacco and alcohol use could be important factors in OVC [38]. As this is the first study comparing fascin expression between OSCC and OVC, further studies are needed with a larger sample size and investigating the mentioned factors and utility of fascin for prognosis in OVC. Our OSCC samples were mostly grade 1, and we have a few numbers of grade 3 OSCC. We recommend that a greater number of grade 3 OSCC be used in future studies.

## 5. Conclusions

The results of the statistical test in the present study showed that the percentage and the intensity of staining are higher in the OSCC, VC, and control groups, respectively. Also, in higher grades of SCC, the percentage and intensity of staining were higher than in lower grades. Finally, it seems that fascin expression has an important role in predicting VC and especially SCC. Also, it may be an appropriate therapeutic target for therapy.

## Figures and Tables

**Table 1:** Demographic data and comparison of the area of involvement in patients with oral verrucous carcinoma (OVC) and oral squamous cell carcinoma (OSCC).

Variable	Oral squamous cell carcinoma (OSCC) Mean ± SD or *N* (%)			Oral verrucous carcinoma (OVC) Mean ± SD or *N* (%)	*p*-Value
	Grade I	Grade II	Grade III		
Age	68.0 ± 9.94	56.67 ± 16.23	58.0 ± 24.32	64.54 ± 13.29	0.276^a^
Sex	—	—	—	—	0.123^b^
Female	8 (66.7%)	2 (22.2%)	1 (25%)	13 (59.1%)	—
Male	4 (33.3%)	7 (77.8%)	3 (75%)	9 (40.9%)	—
Area of involvement	—	—	—	—	*p* < 0.001^c^
Lower vestibule	1 (8.3%)	0 (0.0%)	0 (0.0%)	5 (22.7%)	—
Upper vestibule	5 (41.7%)	0 (0.0%)	1 (25.0%)	4 (18.2%)	—
Tongue	3 (25%)	7 (8.77%)	1 (25.0%)	1 (4.5%)	—
Floor of the mouth	0 (0.0%)	0 (0.0%)	0 (0.0%)	5 (22.7%)	—
Lower lip	2 (16.7%)	0 (0.0%)	0 (0.0%)	1 (4.5%)	—
Cheek	0 (0.0%)	1 (11.1%)	0 (0.0%)	4 (18.2%)	—
Palate	0 (0.0%)	0 (0.0%)	1 (25.0%)	0 (0.0%)	—
Gingiva	1 (8.3%)	0 (0.0%)	1 (25.0%)	0 (0.0%)	—
Ridge and lower vestibule	0 (0.0%)	1 (11.1%)	0 (0.0%)	2 (9.1%)	—
Total	12 (100%)	9 (100%)	4 (100%)	22 (100%)	—

^a^ANOVA test.

^b^Chi-square test.

^c^Fisher's exact test.

**Table 2:** Percentage of stained cells in the groups regarding OSCC histological classification.

Percentage of stained cells	Oral squamous cell carcinoma (OSCC) *N* (%)			Oral verrucous carcinoma (OVC) *N* (%)	Control *N* (%)	*p*-Value
	Grade I	Grade II	Grade III			
1 (5%–25%)	0 (0.0%)	0 (0.0%)	0 (0.0%)	0 (0.0%)	2 (20%)	*p* < 0.001^a^
2 (25%–50%)	0 (0.0%)	0 (0.0%)	0 (0.0%)	5 (22.73%)	6 (60%)	—
3 (50%–75%)	5 (41.67%)	1 (11.11%)	1 (25%)	5 (22.73%)	2 (20%)	—
4 (>75%)	7 (58.33%)	8 (88.89%)	3 (75.0%)	12 (54.55%)	0 (0.0%)	—
Total	12 (100%)	9 (100%)	4 (100%)	22 (100%)	10 (100%)	—

^a^Kruskal–Wallis test.

**Table 3:** Percentage of stained cells and intensity of staining in the three groups.

	Oral squamous cell carcinoma (OSCC) *N* (%)	Oral verrucous carcinoma (OVC) *N* (%)	Control *N* (%)	*p*-Value
Intensity of staining				
1 (weak)	1 (4%)	8 (36.36%)	5 (50%)	*p* < 0.001^a^
2 (moderate)	12 (48%)	13 (59.09%)	5 (50%)	—
3 (severe)	12 (48%)	1 (4.55%)	0	—

Total	25 (100%)	22 (100%)	10 (100%)	—

Percentage of stained cells				*p* < 0.001^a^
1 (5%–25%)	0	0	2 (20%)	—
2 (25%–50%)	0	5 (22.73%)	6 (60%)	—
3 (50%–75%)	7 (28%)	5 (22.73%)	2 (20%)	—
4 (>75%)	18 (72%)	12 (54.55%)	0	—

Total	25 (100%)	22 (100%)	10 (100%)	—

^a^Kruskal–Wallis test.

**Table 4:** Intensity of staining in the groups regarding OSCC histological classification.

Intensity of staining	Oral squamous cell carcinoma (OSCC)			Oral verrucous carcinoma (OVC)	Control	*p*-Value
	Grade I	Grade II	Grade III			
1 (weak)	0 (0.0%)	1 (11.11%)	0 (0.0%)	8 (36.36%)	5 (50%)	*p* < 0.001^a^
2 (moderate)	9 (75%)	2 (22.22%)	1 (25.0%)	13 (59.09%)	5 (50%)	—
3 (severe)	3 (25.0%)	6 (66.67%)	3 (75.0%)	1 (4.55%)	0	—
Total	12	9	4	22 (100%)	10 (100%)	—

^a^Kruskal–Wallis test.

**Table 5:** Comparison of the literature findings in the discussion.

Key finding	Author	Year	Results
Increase of fascin expression in tumors	Shimamura et al. [25]	2011	Higher fascin expression was reported in dysplasia and Oral CIS compared to benign diseases. Fascin expression was observed in all invasive SCC samples.
	Zhang et al. [26]	2006	Fascin expression increases progressively from normal epithelium to invasive esophageal SCC.
	Papaspyrou et al. [18]	2014	Higher fascin levels in tumor tissues were observed.
	Present study		A significant difference in the intensity of staining and percentage of stained cells, which decreased from samples with higher grade OSCC to samples with OVC and control samples, which confirmed the increase in fascin protein expression in OSCC patients.

Weak expression of fascin in tumors	Alam et al. [10]	2012	25.19% of cases had no fascin, 41.98% had weak expression, and 32.28% had increased expression.
	Lee et al. [11]	2007	20 of 40 OSCC samples showed negative to low expression of fascin.

No association between fascin expression and prognostic factors	Zhang et al. [26]	2006	No significant association was observed between fascin expression and prognostic factors.
	Öztürk et al. [28]	2022	No significant association between fascin expression and prognostic factors.

Association of fascin expression and prognosis	Papaspyrou et al. [18]	2014	A longer median overall survival observed in the group with low fascin expression (54 months) compared to the high fascin group (38 months), but the difference was not statistically significant.
	Wu et al. [27]	2010	Lower overall and disease-free survival rates with high fascin expression.
	Lee et al. [11]	2007	High fascin expression was associated with tumor recurrence and overall patient survival.

Fascin expression in normal epithelium	Present study		In our study, the staining intensity score was 2 in 5 samples out of 10 control samples.
	Shimamura et al. [25]	2011	Fascin is not expressed in the normal oral epithelium or only weakly expressed in basal cells.

Intensity and percentage of fascin	Chen et al. [20]	2007	Intensity and distribution of fascin expression were correlated with tumor size and clinical classification.
	Present study		Higher intensity and percentage of staining were observed in most of the cases with higher grades of OSCC.

## Data Availability

The datasets used and/or analyzed during the current study are available from the corresponding author on reasonable request.

## Nomenclature

OSCC: Oral squamous cell carcinoma
OVC: Oral verrucous carcinoma
